# Don’t sweat the small stuff: a conversation about nanosafety

**DOI:** 10.3389/ftox.2023.1254748

**Published:** 2023-08-24

**Authors:** Bengt Fadeel, Phil Sayre

**Affiliations:** ^1^ Institute of Environmental Medicine, Karolinska Institutet, Stockholm, Sweden; ^2^ nanoRisk Analytics LLC, Auburn, CA, United States

**Keywords:** nanosafety, test guidelines, advanced materials, nanomaterials, lessons learned

## Abstract

Bengt Fadeel and Phil Sayre discuss lessons learned with respect to the safety assessment of nanomaterials, and provide a perspective on current and future challenges.

BF: Phil, thank you for taking the time to discuss nanosafety. I suppose that this is not the first time, nor will it be the last, although it seems to me that some questions are merely repackaged for consumption by new generations of students and scientists. For instance, the definition of a nanomaterial, (Joint Research Centre, 2023) something we used to fret over for a very long time *versus* the “smart” or advanced materials that we are faced with today. I am not so sure about the so-called smart materials, but I imagine that advanced materials are novel materials purposefully designed to show improvements of properties or functions when compared to conventional materials. However, it seems in some sense a circular argument to say that a nanomaterial is nano-sized or that advanced materials are advanced; perhaps the definition of advanced materials should consider safety upfront (in other words, safety as an integral part of the definition of “advanced”). But the point is: no matter how smart or advanced the material, toxicologists and regulators have to be smarter. New materials are, by their very nature, a moving target, and we need to be smart when it comes to the safety assessment of all these new (nano) materials.

PS: Thanks, Bengt. My perspective may be a bit different from yours, as I am largely focused on data needs for regulatory approval of commercial nanomaterials in the United States (given my current work in advising companies with respect to nanomaterial risk assessment, and my previous work on nanomaterials both at the EPA and FDA). I do not know that advanced materials are the highest priority at the present time. Large-scale production processes for other traditional nanomaterials pose equally large challenges due to a lack of both mammalian and ecotoxicity hazard information that would be accepted by regulatory authorities. This lack of data risks holding up new technologies, for instance, the development of more efficient batteries that will allow us to move away from fossil fuels, thereby reducing global warming. The current discourse on safety and sustainability needs to consider the bigger picture—not only the potential risks of introducing new nanomaterials on the market, but also the risk of not introducing new materials or new technologies that could help build a greener and cleaner future. Interestingly, a new proposal has been put forth in the United States House of Representatives to amend the Toxic Substances Control Act (TSCA) to allow for more rapid approvals of new chemicals related to critical energy needs in the United States. The proposal for the new bill recommends considering the economic, environmental, and societal benefits of new energy-related chemical(s) along with potential risks when making decisions.

BF: Can you name any specific classes of materials that we should be concerned with?

PS: With the advent of electric cars, there is a pressing need for more efficient lithium-ion battery designs. Current generation batteries are augmented with graphite, but many commercial-scale manufacturers, at least in the United States, are trying to incorporate carbon nanotubes or graphene to push batteries to the next level of performance. However, while data certainly exist on aquatic toxicity, our inquiries to the EPA suggest that there are insufficient data to establish an ecotoxicity level of concern for any CNT. In addition, there are a very limited number of OECD-compliant (OECD, 2018) subchronic inhalation tests for CNTs, and only one chronic inhalation test published in the open literature that has been performed in accordance with OECD standards. (Kasai et al., 2016). The limited amount of acceptable inhalation test results for CNTs (World Health Organization, 2017) often leads to a “no release of (CNT) dust” restriction in workplace environments by the US EPA under TSCA. These inhalation tests are expensive to conduct, involve the use of animals, and data from these tests—even if considered acceptable to regulators—may not be accepted for other CNTs apart from those tested. Better inhalation toxicity data, that would lead to some discrimination between more and less potent CNTs with respect to inhalation toxicity would potentially allow for increased exposures for some CNTs. This would decrease the costs of engineering and PPE controls, allow for a more comfortable work environment, and still be protective of human health. The less costly yet protective restrictions on manufacturing and use would, in turn, enable more competitive production of batteries for electric vehicles.

BF: Graphene, an atomically thin material, has been suggested as an exemplar of advanced materials. Graphene was discovered in 2004, so it certainly qualifies as a new material (or class of materials), but is it advanced? Perhaps it depends on the application (Ferrari et al., 2015). Could you elaborate a bit on graphene from a regulatory horizon?

PS: Similarly, for graphene, it is my understanding that the EPA has insufficient data from acceptable regulatory tests to establish a toxicity level of concern. Contrast this EPA position with the conclusions of the recent study on graphene and other 2D materials commissioned by the European Chemicals Agency (ECHA) which concluded that there are plenty of data already in the scientific literature (European Chemicals Agency, 2022). The discrepancy between the inability of regulators to identify acceptable studies on the (eco) toxicity of graphene, and the conclusion of the latter report that there are extensive data in the open literature, can only be true if the data in the open literature do not meet OECD standards for nanomaterial testing and are therefore not reliable and relevant enough to support regulatory conclusions. This example highlights the importance of producing data to support regulatory decision making.

BF: The EU-funded Graphene Flagship has made great strides in terms of evaluating human health and environmental safety of graphene and other 2D materials, and bringing all this information to the attention of the regulators is a key priority. Overall, I do see a lot of improvement in the way in which nano (eco) toxicology is done, and there is certainly an awareness of the critical importance of carefully characterizing the test materials. In fact, the scientific community is discussing how to improve the quality of the research that is performed, with a call for minimal reporting requirements in biotechnology and toxicology literature (Faria et al., 2018). Essentially, this boils down to three fundamental aspects of any “nano-bio” experiment, namely, the 3 M:s (materials, methods, and models). Thus, details on the nanomaterials and their behavior in the relevant test medium are certainly required, and detailed information on the assays used (and whether the test materials show interference with the assay), and, finally, the model system, that is, the cell type or animal model used. But I would argue that it is not enough to report on materials, methods, and models. We should also consider: when is a model a good model? For a clinician, the ultimate model is the patient, but the same is not true for a toxicologist. Therefore, we are left with models, but why do we choose to work with a certain model? Perhaps because a particular model is recommended for use according to validated protocols, such as the test guidelines (for chemicals) issued by the OECD? Or we may simply choose to work with a particular model for its availability or simplicity or ease of handling or low cost or reproducibility—or all of the above.

PS: There are ongoing and concerted efforts to address the validity of existing OECD test guidelines with respect to nanomaterials under the umbrella of the so-called Malta Initiative. The EU-funded project ProSafe (“Promoting the implementation of safe-by-design”) reviewed the output of several national and international projects some 5 years ago and concluded that several new tools to enable regulatory risk assessment of nanomaterials are available or would become available in the near future. But it was also noted that nanomaterials are “difficult substances” to assess.

BF: Indeed. However, I find that we are sometimes trying too hard to adapt test methods developed for traditional chemicals to the evaluation of nanomaterials. Let me try to explain what I mean: after 10 or 15 years of nanotoxicological research aimed at investigating the impact of engineered nanomaterials on human health and the environment, it seems that we are still far from understanding and controlling the hazard of these materials (Krug, 2014). This could be due, in part, to the fact that nanomaterials display considerable variability with respect to their physicochemical properties, but it may also have something to do with the fact that the assays or test protocols that are used for the evaluation of nanomaterials have been developed with ‘traditional’ chemicals in mind, whereas nanomaterials are not small molecules, (Stark, 2011) even though they most certainly are chemical substances (and should be regulated as such). If a nanomaterial cannot be dispersed in aqueous medium then it is very difficult to study in a biological system such as a cell culture model, and we therefore devise protocols for material dispersion where the test materials are mixed with proteins or lipids or pluronic surfactants (amphiphilic copolymers) followed by sonication to “force” the materials into suspension, but are the outcomes of these tests reflective of the hazardous properties of the nanomaterial itself or have we overlooked the nano-ness of the materials? To resolve this conundrum, we may need new protocols more suited to nanomaterials, but such protocols must still be attuned to the relevant endpoints.

PS: I agree that traditional tests developed for organic chemicals have shortcomings when applied to nanomaterials. OECD member countries recognized that some of the specialized guidance for poorly soluble substances would apply to nanomaterials. However, it became evident that there are many other factors that need adjusting when addressing nanomaterials. Therefore, OECD and its associated member countries have developed several new protocols for characterizing physicochemical properties of nanomaterials, and for the ecotoxicity and mammalian toxicity testing of nanomaterials, and updated other guidelines (e.g., OECD TG 413 for subchronic inhalation toxicity) to specifically accommodate nanomaterials. The availability (and use) of harmonized tools for the regulatory testing and assessment of nanomaterials is of huge importance as we go forward. In terms of specific regulatory acceptance of data, OECD member country regulators are bound to accept data conducted in accordance with OECD test guidelines and guidances. Therefore, development of any new “model” as you noted above is most easily accepted by regulators, along with the data generated, if the models (for both hazard and exposure testing) are accepted by OECD. There have been a few recent research initiatives to evaluate existing OECD test guidelines with respect to their applicability to nanomaterials, including the H2020 project NanoHarmony which seeks to develop a framework for testing of nanomaterials to support regulation, and it is my conviction that such programs should be strongly supported as they not only address the development of high-priority OECD test guidelines (TGs) and guidance documents (GDs) in conjunction with OECD, but they also seek to streamline the TG and GD acceptance process.

BF: Indeed, fit-for-purpose test methods for regulatory compliance are needed, (Bleeker et al., 2023) and the Malta Initiative as you mentioned provides an example of how such efforts can be organized through a partnership between industry, academia, and government institutions. In particular, academic scientists may “test the tests” with respect to their applicability to nanomaterials, but I am not totally convinced that academic laboratories should do the actual testing; it befalls companies to provide information on nanoforms of substances that are subject to registration under the regulation on registration, evaluation, authorisation and restriction of chemicals (REACH). The European Commission recently presented a framework to define safe-and-sustainable-by-design criteria for chemicals and materials in line with the European Chemicals Strategy for Sustainability (CSS). The framework (Joint Research Centre, 2022) adopts a hierarchical approach in which safety aspects are considered first, followed by environmental, social, and economic aspects. But the test methods commonly used for safety assessment should also be considered. Is it sustainable to test every new (nano) material using animal models, or are we finally ready to make the transition towards alternative test methods, using traditional rodent or large animal models as a last resort? I am sure you recall our recent ‘pandemic’ discussion on the R.I.P. guidelines for nanotoxicity testing (Fadeel and Sayre, 2021). Perhaps these are not guidelines so much as an attempt to encapsulate the salient features of a sound approach to testing: 1) R = relevant (realistic *in vitro* and/or *in vivo* models that deliver results that are relevant for risk assessment); 2) I = integrated (as in integrated or tiered approaches to safety assessment, starting with acellular tests and *in vitro* models); 3) P = predictive (i.e., *in vitro* results that are predictive of *in vivo* outcomes, and results that allow for the grouping of nanomaterials on the basis of predictive screening and/or *in silico* modelling, e.g., structure-activity relationships or SARs).

PS: Sure, but remember that even 10–15 years ago when “simple” nanomaterials were of concern, promises were made to generate data, but little to no regulatory data were generated. I see the potential of accelerating regulatory decisions via interpolation between analogs. The problem is that there is insufficient data across a category of closely aligned nanomaterials including many of those being produced in high volumes, such as carbon nanotubes. Regulatory authorities could help by putting together available hazard data, without revealing confidential information, to better allow grouping, and promote the use of other predictive tools.

BF: I sense your frustration, and you certainly have great insights when it comes to the inner workings of regulatory authorities while I am focused more on the fundamental mechanisms of toxicity. But I believe that there is now enough research (which is not necessarily the same as regulatory data) to at least draw some conclusions as we go forward. This is why I am not convinced that all carbon nanotubes should be categorically banned just because certain MWCNTs were shown to cause asbestos-like pathogenicity. We should not throw out the baby with the bathwater; after all, we have learned over the past decade or more that not all carbon nanotubes are alike: some are short and tangled and some are long and rigid, and this makes a big difference in terms of how the human body handles these materials. Moreover, we now know that some carbon nanotubes are biodegradable meaning that these materials are not biopersistent like asbestos fibers (Bhattacharya et al., 2016). My concern is that we may be repeating the same story all over again with the graphene-based materials; again, not all GBMs are alike, chemically or from a toxicological point-of-view, and regulators need to acknowledge these differences.

PS: I agree that all carbon nanotubes are not equivalent: we learned early on that rigid MWCNTs such as Mitsui-7 are more potent in subchronic inhalation studies in rodents, as opposed to less rigid MWCNTs that present as bundles of tangled fibers in similar *in vivo* subchronic inhalation studies. However, we need more data from such *in vivo* subchronic rodent studies that are OECD-compliant to flesh out the differences in toxicity: factors such as the number of walls, presence of catalysts, and other physicochemical properties should be examined. These data should be leveraged by using other available toxicity data from *in vitro* studies which are judged as reliable (in terms of the test protocols as well as the physicochemical characterizations of the materials). Grouping approaches, advocated for by ECHA and recently elaborated by the H2020 program GRACIOUS and others, should be used to build a convincing case for ranking the pulmonary toxicity of various CNTs once these data are available (Murphy et al., 2022). There may also be additional reliable and relevant data from subchronic inhalation and other confidential studies that are held by EPA and ECHA. Indeed, there may be ways for regulatory authorities to redact the data and provide at least trend information on pulmonary toxicity and key physicochemical parameters without disclosing confidential information. This has already been done by the US EPA: the ECOSAR algorithms both protect confidential business information and allow aquatic toxicity to be estimated (and accepted by EPA) based on certain chemical characteristics, by using algorithms that associate specific chemicals with the toxicity data that is held at the EPA. EPA put these data together to assist both EPA estimations of toxicity, and industry estimates. Hence, one can run ECOSAR, given key chemical characteristics, in an early safe-by-design evaluation process also to avoid ecotoxic candidate chemicals for commercialization. EPA and ECHA, working in conjunction with OECD member countries and industry, could assist in building consortia of companies to perform critical testing that would allow grouping of nanomaterials (such as graphene-based materials, and carbon nanotubes and nanofibers) that would be accepted by regulators who assess the potential adverse health and ecological effects of these materials. OECD attempted to do exactly this sort of testing with representative nanomaterials some years ago. Now that better test methods are available, such an approach should be pursued once more.

BF: Coming back to my initial statement, we need to be smart when it comes to the safety assessment of nanomaterials and other sophisticated materials. High-throughput screening (HTS) approaches, using *in vitro* assays reflective of relevant endpoints—meaning endpoints corresponding to *in vivo* outcomes—may prove useful, and omics-based systems biology approaches are also being explored, though the two approaches are principally very different. HTS essentially means that large numbers of nanomaterials are screened using a few (simple) assays, while omics approaches typically refer to the exploration of a small set of nanomaterials with respect to a large number of variables (i.e., all the transcripts, proteins, or metabolites of a system, where the system could be a single cell type or an entire organism). We need to recognize this distinction because we cannot perform “omics” on each and every nanomaterial if the objective is to conduct risk assessment. On the other hand, systems biology approaches can yield novel biomarkers, and may also enrich adverse outcome pathway (AOP) descriptions (Halappanavar et al., 2020). The FP7 project NANOSOLUTIONS serves as a prime example wherein more than 30 nanomaterials were screened with respect to omics “signatures” and the identified mRNA-based biomarkers were found to predict the hazard potential of nanomaterials more reliably than the intrinsic physicochemical properties alone (Fortino et al., 2022).

PS: Yes, I also see the enrichment of AOPs as a more near-term (feasible) goal, at least for determining modes of action and setting further testing priorities. HTS, using an array of simple assays such as *in vitro* and acellular assays, also seems feasible—consider the EPA’s ToxCast (“toxicity forecasting”) program where both organic chemicals and a few nanomaterials were tested. Omics, on the other hand, seems a bit further off in terms of its general use, although such approaches may be considered for chemicals or nanomaterials where several modes of action need to be prioritized for further testing, provided that sufficient funding is allocated to support such testing. All of these approaches, therefore, are useful tools for setting priorities for further testing using currently accepted regulatory protocols.

BF: In closing, I think that you would agree that nanosafety research has come a long way in the last 15 years—not only in terms of how the research is conducted, with close attention to the characterization of the materials, and to the relevance of the model, but also in terms of evolving a blueprint for collaborations that span many different scientific disciplines, and bring academia, industry, and regulators together to address the common goal of making sure that nanomaterials are safe and sustainable. However, as you pointed out, we need more OECD-compliant data to support risk assessment of nanomaterials, and this is certainly an urgent priority (OECD, 2013). Finally, it bears mentioning that “safety” is not an intrinsic material property, and there is no material that is completely without risk. Instead, we as a society need to establish what is “safe enough”, and that is not a decision that can be taken by toxicologists alone, or by regulators alone.

## Further reading

Amorim, M. J. B., Peijnenburg, W., Greco, D., Saarimäki, L. A., Dumit, V. I., Bahl, A., et al. (2023). Systems toxicology to advance human and environmental hazard assessment: A roadmap for advanced materials. Nano Today 48, 101735. 10.1016/j.nantod.2022.101735.

Chen, C., Leong, D. T., and Lynch, I. (2020). Rethinking nanosafety: Harnessing progress and driving innovation. Small 16 (21), e2002503. 10.1002/smll.202002503.

Drasler, B., Sayre, P., Steinhäuser, K. G., Petri-Fink, A., and Rothen-Rutishauser, B. (2017). In vitro approaches to assess the hazard of nanomaterials. NanoImpact 8, 99–116. 10.1016/j.impact.2017.08.002

Fadeel, B. (2022). Nanomaterial characterization: Understanding nano-bio interactions. Biochem. Biophys. Res. Commun. 633, 45–51. 10.1016/j.bbrc.2022.08.095.

Gottardo, S., Mech, A., Drbohlavov, J., Małyska, A., Bøwadt, S., Riego Sintes, J., et al. (2021). Towards safe and sustainable innovation in nanotechnology: State-of-play for smart nanomaterials. NanoImpact 21, 100297. 10.1016/j.impact.2021.100297.

Hjorth, R., van Hove, L., and Wickson, F. (2017). What can nanosafety learn from drug development? The feasibility of "safety by design". Nanotoxicology 11 (3), 305–312. 10.1080/17435390.2017.1299891.

Krug, H. F. (2018). The uncertainty with nanosafety: Validity and reliability of published data. Colloids Surf. B Biointerfaces 172, 113–117. 10.1016/j.colsurfb.2018.08.036.

Steinhäuser, K. G., and Sayre, P. G. (2017). Reliability of methods and data for regulatory assessment of nanomaterial risks. NanoImpact 7, 66–74. 10.1016/j.impact.2017.06.001.

Teunenbroek, T. V., Baker, J., and Dijkzeul, A. (2017). Towards a more effective and efficient governance and regulation of nanomaterials. Part Fibre Toxicol. 14, 54. 10.1186/s12989-017-0235-z.

Valsami-Jones, E., Cassee, F. R., and Falk, A. (2022). From small to clever: What does the future hold for the safety and sustainability of advanced materials? Nano Today 42, 101364. 10.1016/j.nantod.2021.101364.

## Author biographies

BF is a Full Professor of Medical Inflammation Research at Karolinska Institutet in Stockholm, and Vice Chairman of the Institute of Environmental Medicine. He received his M.D. and Ph.D. at Karolinska Institutet, and board certification at the Karolinska University Hospital. He is a Fellow of the Academy of Toxicological Sciences, and chair of the steering group of the national nanosafety platform, SweNanoSafe. He has been involved in numerous EU-funded nanosafety projects as well as the Graphene Flagship (2013-2023), and he has published 290 original articles and reviews in peer-reviewed journals.

PS is an independent consultant with almost 30 years of experience in the environmental field. His main focus is on the regulatory risk assessments and associated test and data requirements for commercial approval of nanomaterials and genetically engineered microorganisms. He previously worked as a Senior Scientist and Associate Division Director for the U.S. Environmental Protection Agency (EPA)’s Risk Assessment Division, where he was involved in the health and ecological assessments of over 160 nanomaterial submissions to the EPA for commercialization under TSCA. He has also served as a consultant to the OECD WPMN, and as an advisor to several EU-funded projects.
